# Randomization in Laboratory Procedure Is Key to Obtaining Reproducible Microarray Results

**DOI:** 10.1371/journal.pone.0003724

**Published:** 2008-11-14

**Authors:** Hyuna Yang, Christina A. Harrington, Kristina Vartanian, Christopher D. Coldren, Rob Hall, Gary A. Churchill

**Affiliations:** 1 The Jackson Laboratory, Bar Harbor, Maine, United States of America; 2 Gene Microarray Shared Resource, OHSU Cancer Institute, Oregon Health and Science University, Portland, Oregon, United States of America; 3 Pulmonary Sciences and Critical Care Medicine University of Colorado Health Sciences Center, Denver, Colorado, United States of America; 4 Center for Array Technologies, Department of Microbiology, University of Washington, Seattle, Washington, United States of America; Victor Chang Cardiac Research Institute, Australia

## Abstract

The quality of gene expression microarray data has improved dramatically since the first arrays were introduced in the late 1990s. However, the reproducibility of data generated at multiple laboratory sites remains a matter of concern, especially for scientists who are attempting to combine and analyze data from public repositories. We have carried out a study in which a common set of RNA samples was assayed five times in four different laboratories using Affymetrix GeneChip arrays. We observed dramatic differences in the results across laboratories and identified batch effects in array processing as one of the primary causes for these differences. When batch processing of samples is confounded with experimental factors of interest it is not possible to separate their effects, and lists of differentially expressed genes may include many artifacts. This study demonstrates the substantial impact of sample processing on microarray analysis results and underscores the need for randomization in the laboratory as a means to avoid confounding of biological factors with procedural effects.

## Introduction

The gene expression microarray has become a ubiquitous tool in modern biology resulting in substantial accumulation of data in public repositories [1]. Researchers now routinely combine or compare results from different studies. This practice raises concerns about the reliability and reproducibility of microarray data that have been generated across multiple laboratories. Several studies have been conducted to compare performance across different gene expression platforms and laboratories [2]–[5]. These studies have generally concluded that, although absolute expression levels may differ, there is a substantial concordance of results obtained. While these findings provide confidence in microarray technology, it is important to be aware that this positive message was based on comparisons of the best-performing laboratories [2] or on small sets of top ranked genes [3]. As we demonstrate here, there is still cause for healthy skepticism regarding the reproducibility of microarray data.

Studies of the reproducibility of microarray data can vary in scope. Most studies use a common set of samples, but the embedded biological signals can be small or large, and they may or may not include truth standards such as ‘spike in’ RNA or mixtures of RNAs from knockout cell lines [2]. One could look at performance across different platforms, across different laboratories or apply different methods of analysis. We chose to look at the effect of processing samples at different laboratory sites. We employed a common array platform, the Affymetrix GeneChip Mouse 430v2, and used a common set of 16 RNA samples with moderate expression differences.

We collected kidney tissue samples from two male and two female mice from the C57BL/6J strain and from each of three chromosome substitution strains (CSSs) [6], C57BL/6J-Chr1^A/J^, C57BL/6J-Chr6^A/J^ and C57BL/6J-Chr15^A/J^. We will denote these strains as B, A1, A6, and A15 in this paper. Sample were distributed to each of four centers, and one center processed two sets of the 16 samples at different times using different labeling protocols. For simplicity we refer to these as five centers (C1–C5). We selected these strains based on the expectation that differentially expressed genes between the background strain B and each of the CSSs would be enriched for genes on the substituted chromosome. However there are no truth standards so our results reflect the precision but not necessarily the accuracy of the platform. Samples were delivered to each of the sites with the suggestion that they be processed according to standard protocols in a manner typical for that laboratory. Data from each center were provided in the form of CEL files.

To investigate variability among centers, we applied a typical collection of interpretive analysis tools to data from each laboratory and made quantitative comparisons of the results. These analysis tools address the objective of generating and comparing lists of differentially expressed genes, identifying enriched biological pathways that are in common or differ between experiments, clustering the samples by expression pattern, and classifying new samples [7]–[10] using accumulated data.

The efforts of user groups such as The Microarray and Gene Expression Data (MGED) society [1] have enabled the sharing of both primary and procedural data from microarray studies. The value of these resources depends in part on the availability of detailed description of the platforms and procedures used to generate the data. In this study we demonstrate that dramatically different results can be obtained when the same samples are processed in different laboratories. We identify and discuss the procedural origins of some of these differences. This illustrates both the importance and the limitation of current experimental annotation standards.

## Results

### Normalized intensity profiles

**Figure 1:**
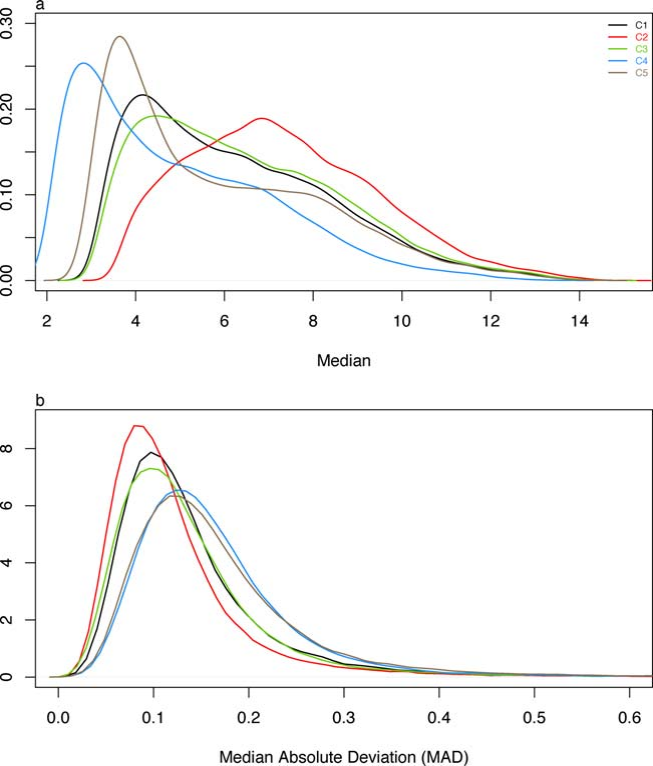
Intensity across centers. Median intensity (a) and Median absolute deviation across centers (b). Samples from each center are individually RMA processed.

We normalized data from each center separately using the Robust Multichip Average method (RMA) [11]. The distribution of normalized intensities is an important diagnostic of microarray data quality. RMA processed intensities can be plotted as smoothed histograms as in **Figure 1a**. The distributions vary dramatically across centers. Centers 2 has the highest median intensity. This was not unexpected because center 2 used the original Affymetrix array labeling protocol incorporating two labeled NTPs (biotin UTP and biotin CTP), and all other centers used a newer labeling protocol that incorporates one NTP (biotin UTP). However, the low intensity of data from centers 4 and 5 is notable. Distributions for centers 1 and 3 show median intensities in the expected range.

Microarray analysis is typically focused on changes in expression level across conditions. Thus differences in intensity between samples are often more important than absolute intensity levels. The median absolute deviation (MAD) across 16 arrays within each center (**Figure 1b**) show that centers 4 and 5 have the highest internal variation and center 2 has the lowest.

As an aside, we normalized the entire set of 80 arrays from all centers together. This reduced the overall intensity differences but created an even greater difference in MAD distributions between centers 4 and 5 compared to the other centers. From the outset of our analysis we can see that there are differences in the distribution of intensity across centers that cannot be removed by normalization procedures. All results described below are based on RMA normalization of data from each center separately.

### Differential expression

**Figure 2:**
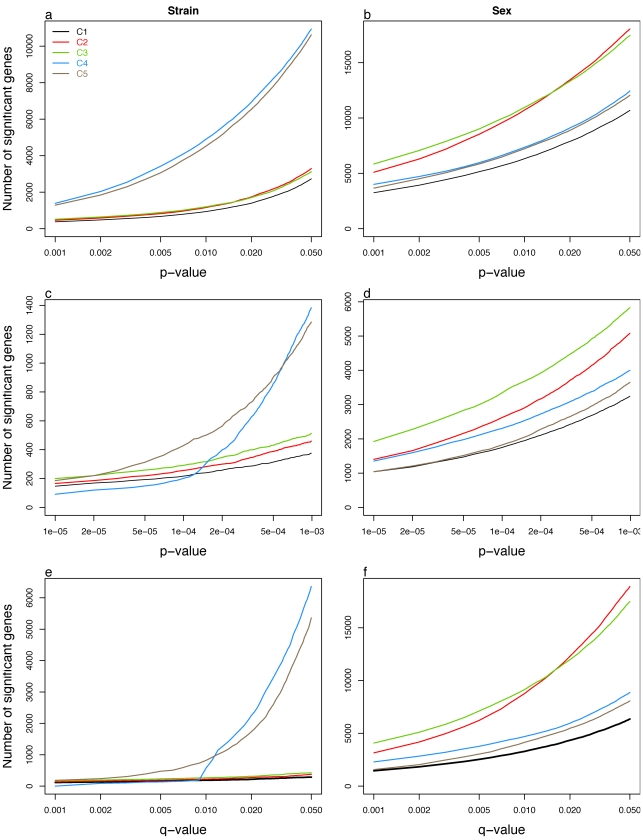
Number of differentially expressed (DE) genes having strain (a, b and c) and sex effect (d, e and f). Number of DE genes selected by p-value (a and b), focusing on up to p-value<0.001 (c and d) and q-value (e and f). Center 4 and 5 have higher number of DE genes when strain effect was tested. However, Center 4 has smaller number of genes than other centers until p-value<2e-04 (panel c), then the number of gets increased, and that causes sudden increase in q-value (panel e). When sex effect is tested, center 2 and 3 show higher number of DE genes than other centers, however the difference is not as big as that of strain effect.

Our experiment includes two biological factors of interest, strain and sex. We constructed statistical tests for the effects of these factors using a linear model that included both factors as main effects with no interaction. Thus tests for strain differences are adjusted for sex and vice versa. For strain differences we consider a test for overall differences as well as each of the pairwise contrasts. Other models and comparisons could be considered but we found these to be sufficient for illustrating the differences among centers. We determined the numbers of genes having significant strain or sex effects using p-value, q-value, and fold-change criteria (**Table 1**; **Figure 2**). There are striking differences in the numbers of differentially expressed genes across centers. For overall strain effects, the numbers of differentially expressed genes from centers 4 and 5 can be as much as five times that of other centers. The same trend is seen for the pairwise strain comparisons with two exceptions; the comparisons of B versus A1 within center 5, and the comparison of A6 versus A15 in center 4 are comparable to the results from centers 1, 2, and 3. Conversely, for sex effects, centers 2 and 3 have the longest lists. In all cases, these differences are the most pronounced when using the q-value criteria and least when fold-change criteria are used.

**Table 1:** Numbers of DE genes having significant sex and strain effects at various p-value, q-value, and fold-change thresholds.

	Threshold	C1	C2	C3	C4	C5
Strain effect						
Overall	0.001	377	462	517	1390	1290
p-value	0.01	939	1160	1200	4907	4522
	0.05	2732	3296	3119	10943	10618
Overall	0.001	116	134	170	0	184
q-value	0.05	188	219	265	571	819
	0.01	285	376	423	6362	5361
	1	135	103	118	216	532
Fold-change	1.5	56	44	52	69	124
	2	29	23	35	34	57
	3	7	6	8	8	10
Pairwise strain effect						
B vs.A1	0.001	223	252	270	458	295
p-value	0.01	648	911	814	2124	989
	0.05	2202	3002	2507	6185	3540
B vs.A6	0.001	119	190	198	1206	417
p-value	0.01	400	703	704	4771	1963
	0.05	1699	2498	2667	10889	6121
B vs.A15	0.001	150	133	123	821	616
p-value	0.01	714	460	542	3068	2765
	0.05	3067	2224	2302	7368	6962
A1 vs.A6	0.001	323	367	463	820	605
p-value	0.01	815	826	1086	2600	2407
	0.05	2591	2129	3005	6390	6883
A1 vs.A15	0.001	284	301	372	1180	1005
p-value	0.01	873	986	927	3424	3728
	0.05	3067	3490	2794	7495	8361
A1 vs.A15	0.001	209	287	254	313	1053
p-value	0.01	641	919	783	1531	4286
	0.05	2377	3344	2552	5493	10188
Sex effect						
	0.001	3250	5099	5842	4003	3663
p-value	0.01	6327	10721	10959	7357	7221
	0.05	10691	18039	17445	12439	12054
	0.001	1452	3149	4078	2292	1568
q-value	0.01	3287	8766	9141	4697	4146
	0.05	6360	18910	17475	8872	8071
	1	313	277	320	347	368
Fold-change	1.5	139	126	139	167	145
	2	86	70	89	110	88
	3	35	31	36	54	40

Fold-change is based on the log_2_ transformation.

To further investigate the effects of the multiple test adjustment on differential gene lists, we looked at the estimated proportions of non-differential genes (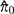), and the false discovery rate [12]–[13] corresponding to fixed p-value thresholds for overall strain and sex effects (**Table 2**). Centers 4 and 5 have much higher estimated proportions of differentially expressed genes (lower 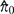) than the other centers. For the sex effect, centers 2 and 3 have somewhat higher estimated proportions of differentially expressed genes. When 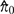 is lower, the q-value corresponding to a fixed p-value is lower. Thus the q-value criteria will tend to exaggerate differences in the list lengths as we have observed.

**Table 2:** The estimated proportion of non DE genes (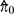), and q-values corresponding to fixed p-values for each center.

		C1	C2	C3	C4	C5
Strain	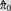	0.96	0.95	0.99	0.42	0.43
	p-value	q-value				
	0.001	0.12	0.09	0.09	0.01	0.02
	0.01	0.46	0.37	0.38	0.04	0.04
	0.05	0.80	0.65	0.72	0.09	0.09
Sex	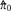	0.70	0.36	0.39	0.53	0.62
	p-value	q-value				
	0.001	0.01	0.00	0.00	0.01	0.01
	0.01	0.05	0.01	0.02	0.03	0.03
	0.05	0.16	0.04	0.05	0.10	0.12

The numbers of DE genes found in common among pairs of centers and among all centers using q-value<0.05 is shown in **Table 3**. Center 4 and 5 have more DE genes and thus share a greater number of DE genes but the proportion of shared genes is only about 17%. On the other hand, centers 1, 2, and 3 have fewer DE genes, and fewer DE genes in common among pairs of centers, but the proportions are higher at about 50%. For the sex effect, centers 2 and 3 share the most common DE genes since both have many DE genes and have proportion in common at about 54%. For the other centers the proportion of common genes is at about 50%, indicating greater consistency across centers for sex effect than for strain effect.

**Table 3:** Number of significant DE genes selected at q-value<0.05, and GO terms enriched in DE genes using Hypergeometric test at p-value<0.01.

	Strain		Sex	
	Num.of Genes	Num.of GO terms	Num.of Genes	Num.of GO terms
C1	285	14	6360	56
C2	376	11	18910	53
C3	426	16	17475	49
C4	6362	50	8872	48
C5	5361	47	8971	55
C1&C2	213	3	5516	10
C1&C3	229	5	5659	14
C1&C4	251	2	4808	22
C1&C5	257	1	4896	27
C2&C3	277	6	12715	14
C2&C4	320	3	7583	13
C2&C5	328	2	6803	6
C3&C4	357	6	7655	17
C3&C5	359	2	6900	14
C4&C5	1672	13	5526	24
All	182	1	4044	2

**Figure 3:**
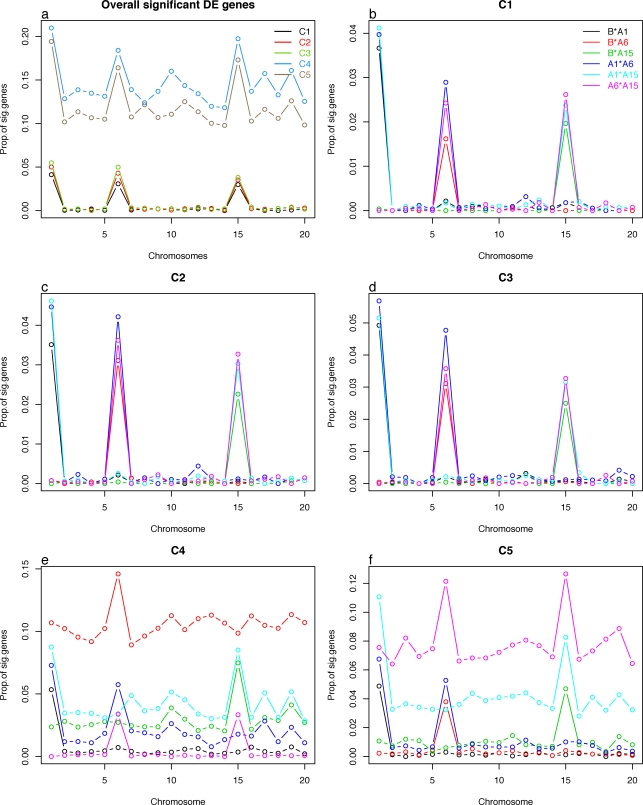
Number of significantly differentially expressed (DE) genes in each chromosome selected by q-value<0.05. Panel a shows the DE genes from the overall test, and Panel b to f show the DE genes from the pairwise strain comparisons from each center. Center 4 and 5 have higher number of DE genes across all chromosomes. Panel e and f show higher number of DE genes in center 4 (B vs. A16) and center 5 (A15 vs. A1 or A6).

To understand the nature of different number of DE genes across center, we studied them by chromosome. Since samples were obtained from chromosome substitution strains, we expected to see enrichment of DE genes on the substituted chromosomes. From the overall and strain pairwise comparisons tests, we selected DE genes by q-value<0.05 and obtained the proportion of significance gene by chromosomes (**Figure 3**). As we expected, higher number of DE genes were found at chromosomes 1, 6, and 15. Center 4 and 5 show the same pattern, except the number of DE genes are higher than other centers across all chromosomes. Pairwise strain comparison show B and A6, and B and A15 strain comparisons have higher number of DE genes in chromosome 6 and 15, respectively, as we expected. However, center 4 and 5 show notably higher number of DE genes in B and A6, and A15 and A1 or A6 comparison, respectively, and we will discuss this later.

**Figure 4:**
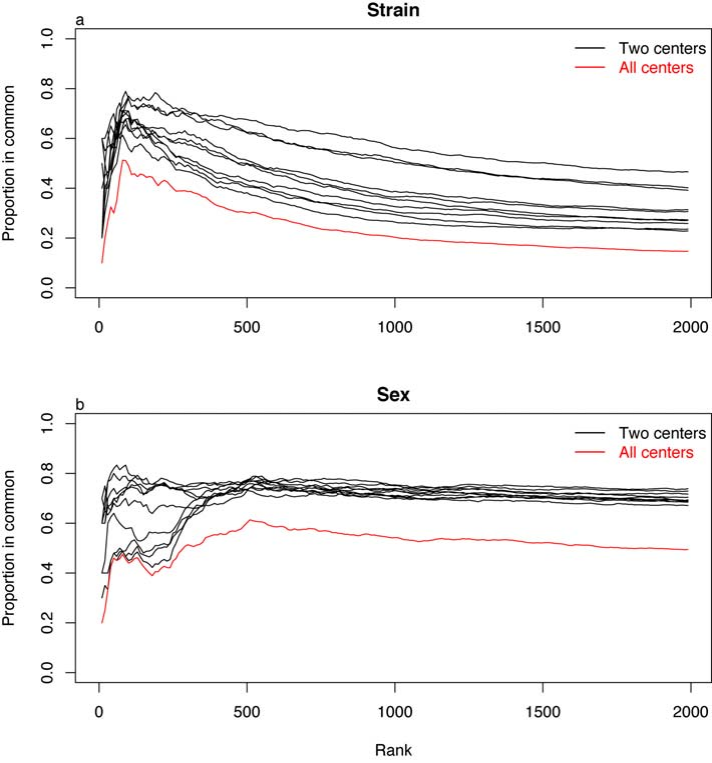
DE genes are selected by rank of Fs test statistics. The proportion of genes shared by centers when strain (a) and sex (b) being tested.

To avoid the problem of comparing lists of very different lengths, we considered genes in rank order by the size of the Fs test statistic or equivalently by p-value. If the rank order of genes is preserved, we might conclude that results are consistent across centers but that p-values are poorly calibrated. We selected lists of various fixed lengths based on rank ordering and calculated the proportion of genes found in common across centers. This type of analysis has been referred to as corresponding at the top (CAT) by Irizarray *et al.* [2] In general, the proportion of genes in common across centers initially increases and then slowly decreases as the list length is increased (**Figure 4**). For the overall strain comparison, the CAT plots peak at a list length of about 70 genes with 60% to 80% pairwise correspondence, and overall correspondence of about 50% in common across all centers. For sex effects, there is some non-monotone behavior in pairwise comparisons. Best peak correspondence is achieved at list lengths of 500 and, this high level of correspondence at 50% to 70% persists for very long lists.

Strong concordance based on rank order indicates that many of the centers are picking the same genes and suggests that calibration of p-values and q-values is contributing to the difference in list lengths. It does not guarantee that the lists are biological in origin. If we had data from only one center, it would be difficult to detect these problems or to determine how many genes are truly differentially expressed.

### Functional analysis

Gene lists established by statistical criteria may vary across centers, but it is possible that they will still support the same biological functions. To test this idea, we selected differentially expressed genes for overall strain and sex effect at q-value<0.05 and identified significant GO terms [14] enriched in the selected genes using Hypergeometric test [15] at p-value<0.01. The numbers of significant GO terms supported by the strain effects lists are much longer for centers 4 and 5 compared to the other centers. There is limited pairwise overlap among the centers and only a single GO term (GO:0048276, the process of gastrulation) in common among all five (**Table 3**). For the sex effects, there are about 50 significant GO terms for each center, and overlap is the best among centers 1, 4 and 5. Two GO terms (GO:0019752, carboxylic acid metabolic process; GO:0006631, fatty acid metabolic process) were found among all five centers.

### Principle components

**Figure 5:**
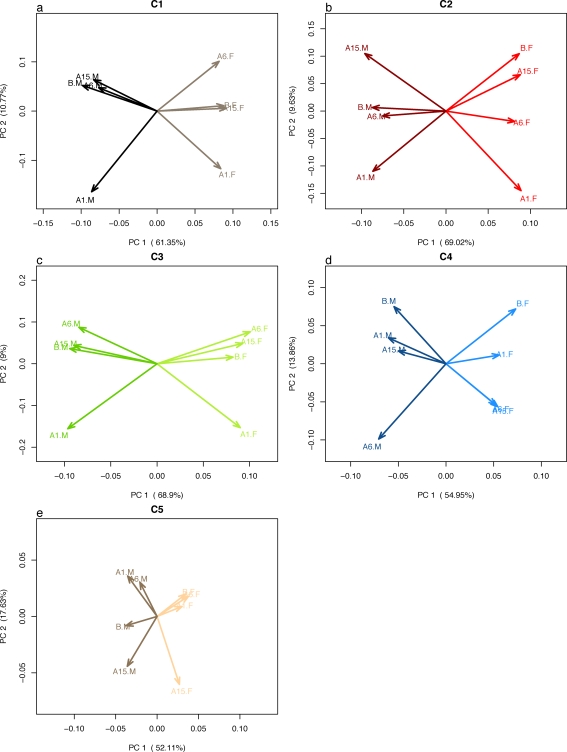
Principal component analysis is applied to data from each center, and first and second principal components are plotted.

Another way to assess the similarity among centers is to identify common features in the major patterns of variation. We applied principal component analysis (PCA) to data from each of the five centers and plot the first vs. second (**Figure 5**) principal components. The first principal component from each center corresponds to the sex effect; it separates male vs. female samples. The second principal component is different for each center. For most centers the second principal component tends to separate strain effects but not in a consistent manner across centers. For instance, second principal components from centers 1 and 3 separate A1 from A6 (or A1 from other centers) but center 5 groups these strains together.

### Classification of samples

In this section we address the problem of combining data across centers for the purpose of classification of samples. We used nonnegative matrix factorization (NMF) method, more specifically the metagene projection procedure as proposed by Tamayo *et al.* [9].

**Figure 6:**
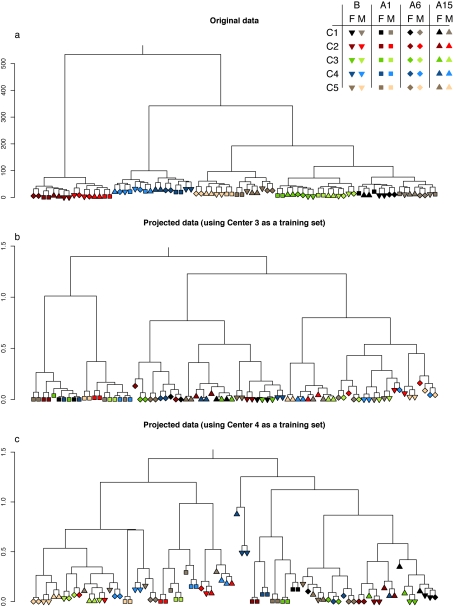
Sample clustering of original data (a). Using center 3 and center 4 as training set, get the H matrix (containing expression level of metagene from each sample), project the metagene to each center and cluster the projected data (b and c respectively).

First we computed a hierarchical clustering of samples using the original data for all centers together (**Figure 6a**). The data is first clustered perfectly by center. Within centers, there is also perfect clustering by sex, and there is a reasonably good pairing of samples of the same strain within sexes. This reflects the relative magnitude of signals as we observed with the PCA.

Next, following Tamayo *et al.* [9], we chose one center as a training set, computed the expression profile of the metagene and weight matrices, and projected the corresponding metagene profiles onto the other four data sets. Clustering of the metagenes obtained using center 3 as the training set (**Figure 6b**) first separates the A1 strain from the other strains. Within A1 it separates sex, and within the other strains it separates sex first then separates strains. Male samples from strains B and A6 are mixed, but in general the separation according to the strain and sex is reasonable. However when center 4 was used as a training set, there is no consistent pattern in the clusters (**Figure 6c**). Clusters derived by training on each of the other centers vary in quality between these two extremes (**Supplementary** **Figure S1**).

This illustrates the risks of combing data or classifying samples using existing data. Although in one case (training on center 3) we were able to extract a biological signal from the combined data, in practice it may be difficult to know when the projection method is effective.

### Batch effects

We demonstrated that lists of differentially expressed genes vary across centers. Here we consider a possible explanation for those differences. Variation in the overall intensity distribution and MAD (**Figure 1**) within each center suggest the differences were generated while the samples were being processed. A retrospective analysis of the laboratory steps used to process the samples revealed that samples were processed together in batches in a manner that reflects some of the differences that we observed. Affymetrix GeneChip arrays are processed in four steps: labeling, hybridization, wash and staining, and scanning. We identified samples that were processed together in each of these steps in each of the centers and identified batch effects that help to explain some of the differences that we observed between centers. Due to personnel and equipment constraints, all samples may not be processed at one time. For instance, one fluidics station used for the wash and staining step, is able to process only up to four samples. The numbers of array that can be hybridized together is constrained along with several other steps in sample processing. Each center resolved these limitations in different ways, and some of these are problematic.

Center 4 hybridized samples in two days; eight samples from B and A1 on the first day and another eight samples from A6 and A15 on the second day. Center 5 hybridized samples over three days; B and A1 on the first, A6 on the second, and A15 on the third day (**Supplementary** **Table S1**). This is consistent with the long lists of differential expressed genes among strain for these two centers. For center 4, the pairwise comparisons between strains B and A1 and between A6 and A15 produced relatively short lists (**Table 1**). For center 5, the B versus A1 comparison is similar to centers 1, 2, and 3 (**Table 1**).

Both centers 2 and 3 stored male arrays at 4 degrees while female samples were washed and stained. These centers have the longest lists of differentially expressed genes between sexes (**Figure 2**). The effect is less than that for the strain comparisons between centers 4 and 5, which may indicate that the batch effects of wash and staining are less dramatic or may it reflect the strength of signal in the sex effect, or both.

Unlike other centers, center 1 randomized the samples before processing. Arrays were hybridized in two batches, one of 12 samples and another with four samples, on the same day. Due to chance in the randomization, the smaller batch included 3 male samples resulting in partial confounding of batch with sex. In all comparisons center 1 has consistently has the shortest list of differentially expressed genes (**Table 1**).

In all cases where processed batches correspond to a biologically interesting feature of the data, we see an increase in the number of apparently differentially expressed genes. For any one center it is not possible to distinguish the effect of batch processing from the biological effect. The magnitude of the batch effect can be significant or negligible, but it cannot be removed from the data without compromising the biological signal.

## Discussion

We have described laboratory to laboratory variation in microarray data that can be attributed to effects of processing samples in batches. The same RNA samples were processed independently five times in four different laboratories using the same Affymetrix GeneChip platform. In centers 2 and 3, female and male samples were processed separately at the washing step, and in centers 4 and 5, samples were hybridized together in groups defined by strain. In all cases, the gene lists corresponding to the confounded factors were substantially longer than gene lists obtained from the other centers where the same confounding was not present. Although in this study we were able to identify the confounding factors, this kind of detailed information about sample processing is not typically available in publicly archived microarray data. Methods to correct for batch effects have been proposed [16], [17] and their benefits have been demonstrated. However when a batch effect is confounded with an experimental factor, correcting for the batch will also effectively remove the biological signal. It is a common practice to organize samples having similar characteristics in groups that are processed together. We strongly recommend against this practice. It is important to identify all potential batch effects in a sample processing pipeline and to assign samples to batches using a randomization mechanism.

With the benefit of hindsight, we might have recommended that in this experiment the samples be processed in each laboratory as follows. The samples should be hybridized in two batches of eight. Each batch should consist of a randomly chosen sample from each of the eight sex-strain pairs. Within each batch the wash and staining should be done on randomly selected sets of four samples, or with random selections balanced across sexes. In addition to avoiding confounding, this strategy has the added benefit that batch can be included as a random effect term in the per-gene ANOVA model to reduce error variation and thus increase power of the design.

We do not intend to convey a message that the microarray data are not reliable. Instead, we wish to highlight the importance of randomization in all laboratory procedures. Careful attention to the potential for confounding effects of these procedures will improve the quality of microarray data and the reliability of analysis results. The effects we observed here primarily impact the length of differentially expressed gene lists, and these are exacerbated by the application of FDR correction [13]. The statistical criterion is correctly identifying genes that differ between groups, but the perturbations that causes these differences represent a mixture of biological and technical effects. In a randomized experiment, technical variation will be balanced across treatment groups, and differentially expressed gene lists will more accurately reflect the biological differences between samples. The laboratories involved in this study have updated their practices to incorporate randomized assignment of samples to processing batches. This can be a challenging problem that requires careful coordination of samples to achieve efficient throughput without compromising data integrity.

## Materials and Methods

### RNA Samples

Kidney tissue samples were collected from two male and two female mice from the C57BL/6J strain and from each of three chromosome substitution strains (CSSs), C57BL/6J-Chr1^A/J^, C57BL/6J-Chr6^A/J^ and C57BL/6J-Chr15^A/J^. In these CSSs, one chromosome derived from the inbred strain A/J has been crossed onto the C57BL/6J background [6]. A total of 16 RNA samples were prepared as described previously [18]. The Animal Care and Use Committee at The Jackson Laboratory reviewed and approved all animal procedures.

### Target labeling and hybridization

All centers used the one-cycle cDNA synthesis/in vitro-transcription (IVT) method recommended by Affymetrix to amplify and label cRNA targets for array hybridization (Affymetrix GeneChip Expression Analysis Technical Manual). Centers 1, 3, 4, and 5 used the IVT reagent kit supplied by Affymetrix while center 2 used the IVT kit supplied by ENZO. Target fragmentation, array hybridization and post-hybridization array processing were performed as per manufacturer's recommendations. However, specific implementation of labeling, hybridization and array processing protocols were according to routine procedures in operation in each center. Several differences in protocol implementation among centers were noted. These include: 1) amount of hybridization solution applied to each array; 2) rotation per minute (rpm) of hybridization oven; and 3) batch design for labeling, hybridization and post-hybridization processing. Details of the protocols used at each center are provided in **Supplementary** **Table S1**.

### Data analysis

Data from each center were provided in the form of CEL files. To investigate variability among centers, we normalized and analyzed data from each center separately.

Preprocessing to remove background signal and to normalize across arrays was carried out using the Robust Multichip Average method (RMA) [11] with software implemented in the R language [19]. To identify differentially expressed genes we fit a general linear model with terms for sex and strain to the data from each gene. We computed the Fs statistic [20] using the MAANOVA package and moderated F-statistic [21] from the LIMMA package, both implemented in R. We also found that the two test statistics, Fs and moderated F, yielded similar results as shown in Opgen-Rhein *et al.* [22] and reported only the results from the Fs statistic here. Nested permutation of sample labels was carried out to obtain p-values for tests of sex and strain effects. Multiple test adjustments were computed using the false discovery rate method [12], [13] as implemented in the q-value program [13]. To explore the biological meaning of the differentially expressed gene lists, we applied a hypergeometric test implemented in the GOstat R package to identify over represented GO terms in the gene lists [15].

Principal Components Analysis (PCA) is used for unsupervised learning. PCA applies a singular value decomposition to the covariance matrix of gene expression data to identify the combination of conditions that explain the greatest variation in the data. PCA was also used by Waring *et al.* [5] to illustrate the reproducibility of microarray data across multiple centers. We applied PCA separately to each center to see if the same components of variation could be identified.

Accumulated data can be used to classify new samples [7]–[10]. We used non-negative matrix factorization (NMF) for classification purpose. NMF partitions data *V* using two non-negative matrices *W* and *H*, *V*∼*WH* where *W* is a weight matrix that indicates how much each gene contributes to each metagene pattern, and *H* contains the expression profiles of the metagenes.

The metagene data can then be used to cluster genes or samples. Among many implementation of NMF, we adopted the metagene projection procedure as proposed by Tamayo *et al.* [9]. In the metagene projection method, a weight matrix *W* obtained from a training set is used to compute the metagene profiles of a target data set (H^*^ = W^−1^ V^*^, where V^*^ is the target data). Tamayo *et al.* [9] show that the projection can correctly cluster samples from cross-platform or even cross-species studies. In our application of this method, we chose one center as the training set, computed the weight matrix, used it to compute a projection for each of the other centers and clustered samples based on the projected metagenes. We repeated this procedure using each center as the training set, and compared the classification results. To compare the performance of this approach, we applied hierarchical clustering to the raw data. Euclidean distance is used to measure the distance, and complete linkage method [23] is used to cluster centers.

## Supporting Information

Figure S1 Hierarchical clustering of H matrices from metagene projection procedure using center 1, 2, or 5 as training sets.
(1.69 MB TIF)

Table S1 Deatil sample processing information.
(0.03 MB XLS)

## References

[1] Brazma A, Robinson A, Cameron G, Ashburner M (2000). One-stop shop for microarray data.. Nature.

[2] Irizarry RA (2005). Multiple-laboratory comparison of microarray platforms.. Nature Methods.

[3] Larkin JE (2005). Independence and reproducibility across microarray platforms.. Nature Methods.

[4] Members of the Toxicogenomics Research Consortium (2005). Standardizing global gene expression analysis between laboratories and across platforms.. Nature Methods.

[5] Waring JF (2004). Inter-laboratory evaluation of rat hepatic gene expression changes induced by methapyrilene.. Environ Health Perspect.

[6] Singer BJ (2004). Genetic Dissection of Complex Traits with Chromosome Substitution Strains of Mice.. Science.

[7] Bullinger L (2004). Use of gene-expression profiling to identify prognostic subclasses in adult acute myeloid leukemia.. N Engl J Med.

[8] Valk PJ (2004). Prognostically useful gene-expression profiles in acute myeloid leukemia.. N Engl J Med.

[9] Tamayo P (2007). Metagene projection for cross-platform, cross-species characterization of global transcriptional states.. Proc Natl Acad Sci USA.

[10] Dudoit S, Fridlyand J, Speed TP (2000). Comparison of discrimination methods for the classification of tumors using gene expression data.. Journal of the American Statistical Association.

[11] Irizarry RA (2003). Summaries of Affymetrix GeneChip probe level data.. Nucleic Acid Res.

[12] Benjamini Y, Hochberg Y (1995). Controlling the false discovery rate: a practical and powerful approach to multiple testing.. Journal of the Royal Statistical Society. Series B.

[13] Storey JD (2002). A direct approach to false discovery rates.. Journal of the Royal Statistical Society Series B.

[14] The Gene Ontology Consortium (2002). Gene Ontology: tool for the unification of biology.. Nature Genet.

[15] Falcon S, Gentleman R (2007). Using GOstats to test gene lists for GO term association.. Bioinformatics.

[16] Leek JT, Storey JD (2007). Capturing heterogeneity in gene expression studies by surrogate variable analysis.. PLoS Genetics.

[17] Seo D, Goldschmidt-Clermont PJ, West M (2007). Of mice and men: Sparse statistical modeling in cardiovascular genomics.. Ann Appl Statist.

[18] Shockley KR, Churchill GA (2006). Gene expression analysis of mouse chromosome substitution strains.. Mamm Genome.

[19] Ihaka R, Gentleman RR (1996). A Language for Data Analysis and Graphics.. Journal of Graphical and Computational Statistics.

[20] Cui X, Hwang JT, Qiu J, Blades NJ, Churchill GA (2005). Improved statistical tests for differential gene expression by shrinking variance components estimates.. Biostatistics.

[21] Smyth GK (2004). Linear models and empirical Bayes methods for assessing differential expression in microarray experiments.. Statistical applications in Genetics and Molecular Biology.

[22] Opgen-Rhein R, Strimmer K (2007). Accurate ranking of differentially expressed genes by a distribution-free shrinkage approach.. Statist Appl Genet Mol Biol.

[23] Hartigan JA (1975). Clustering Algorithms.

