# Supplementary figures and images for: Randomization in Laboratory Procedure Is Key to Obtaining Reproducible Microarray Results
Source: PLoS One. 2008 Nov 14;3(11):e3724. doi: 10.1371/journal.pone.0003724 (PMC2579585; doi:10.1371/journal.pone.0003724)

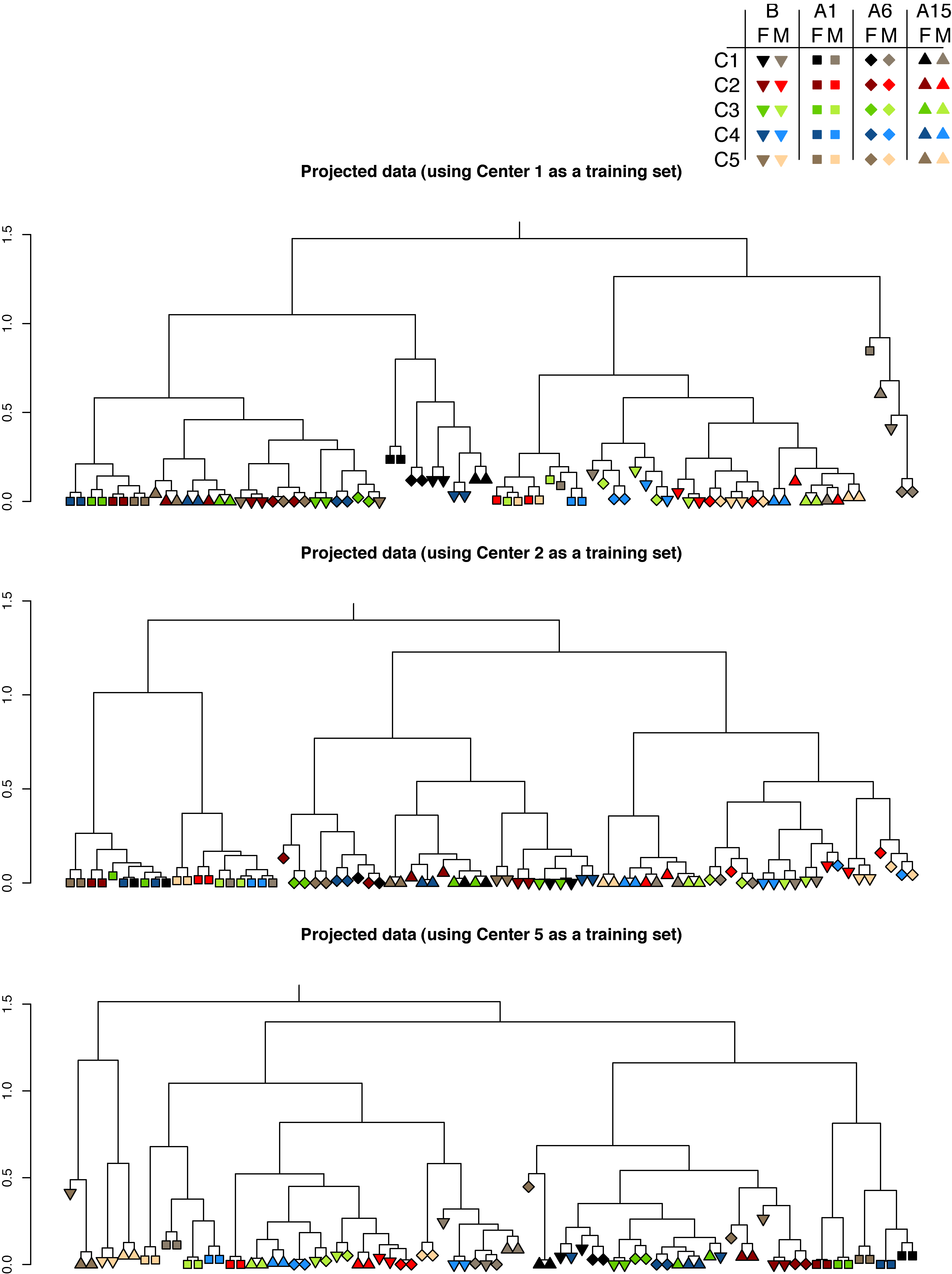

Supplement: Figure S1 — Hierarchical clustering of H matrices from metagene projection procedure using center 1, 2, or 5 as training sets. (1.69 MB TIF) [file pone.0003724.s001.tif]
